# The effects of chemogenetic targeting of serotonin-projecting pathways on L-DOPA-induced dyskinesia and psychosis in a bilateral rat model of Parkinson’s disease

**DOI:** 10.3389/fncir.2024.1463941

**Published:** 2024-11-14

**Authors:** Natalie Lipari, Ashley Galfano, Shruti Venkatesh, Han Grezenko, Ivette M. Sandoval, Fredric P. Manfredsson, Christopher Bishop

**Affiliations:** ^1^Department of Psychology, Binghamton University, Binghamton, NY, United States; ^2^Barrow Neurological Institute, Phoenix, AZ, United States

**Keywords:** PDAP, DREADDs, 5-HT, LID, bilateral, PD

## Abstract

**Introduction:**

Parkinson’s disease (PD) is commonly characterized by severe dopamine (DA) depletion within the substantia nigra (SN) leading to a myriad of motor and non-motor symptoms. One underappreciated and prevalent non-motor symptom, Parkinson’s disease-associated psychosis (PDAP), significantly erodes patient and caregiver quality of life yet remains vastly understudied. While the gold standard pharmacotherapy for motor symptoms Levodopa (LD) is initially highly effective, it can lead to motor fluctuations like LD-induced dyskinesia (LID) and non-motor fluctuations such as intermittent PDAP. One source of these fluctuations could be the serotonergic raphe nuclei and their projections. Serotonin (5-HT) neurons possess the machinery necessary to convert and release DA from exogenous LD. In DA-depleted brain regions these 5-HT projections can act as surrogates to the DA system initially compensating but chronically leading to aberrant neuroplasticity which has been linked to LID and may also contribute to non-motor fluctuations. In support, recent work from our lab established a positive relationship between LID and PDAP in parkinsonian rats. Therefore, it was hypothesized that normalizing 5-HT forebrain input would reduce the co-expression of LID and PDAP.

**Methods:**

To do so, we expressed 5-HT projection specific inhibitory designer receptor exclusively activated by designer drugs (DREADDs) using Cre-dependent AAV9-hM4di in tryptophan hydroxylase 2 (TPH2)-Cre bilaterally 6-OHDA-lesioned rats. Thereafter we used the designer drug Compound 21 to selectively inhibit 5-HT raphe projections during LD treatment to modulate the expression of PDAP, assayed by prepulse inhibition (PPI) and LID, quantified by the abnormal involuntary movements (AIMs) test.

**Results:**

Our results suggest that chemogenetic inhibition of 5-HT raphe-projecting cells significantly reduces LID without affecting stepping ability or established sensorimotor gating deficits

**Discussion:**

Overall, this study provides further evidence for the complex influence of 5-HT raphe-projecting neurons on LD’s neurobehavioral effects.

## Highlights

- There is a positive relationship between dyskinesia severity and sensorimotor gating dysfunction in a bilateral 6-OHDA model of PD.
- Chemogenetic inhibition of 5-HT forebrain projections differentially mediated PDAP and LID supporting unique changes in 5-HT neuroplasticity in PD that could be leveraged for novel treatment approaches.

## 1 Introduction

Parkinson’s Disease (PD) is a prevalent neurodegenerative disorder characterized by progressive dopamine (DA) loss in the substantia nigra pars compacta (SNc; Ehringer and Hornykiewicz, 1998; Alexander, 2004; Davie, 2008; Giguère et al., 2018) leading to motor deficits and a multitude of non-motor symptoms (Ou et al., 2021). While non-motor symptoms have been argued to erode quality of life more than motor features, they are less addressed when implementing therapeutic interventions (Martinez-Martin et al., 2009; Chaudhuri and Odin, 2010).

Parkinson’s disease associated psychosis (PDAP) is a prevalent non-motor symptom characterized by progressively worsening visual hallucinations. First, they manifest as false sensory perceptions without the trigger of a physical stimulus, later proceeding to illusions, the incorrect and distortion of actual sensory stimuli, and lastly, delusions, false idiosyncratic beliefs that are resistant to reasoning (Forsaa et al., 2010; Schneider et al., 2017; Chang and Fox, 2016; Han et al., 2018; Zahodne and Fernandez, 2010). There are several risk factors associated with PDAP such as disease progression, cortical and limbic atrophy, and DA replacement therapy (Burton et al., 2004; Forsaa et al., 2008, 2010; Chang and Fox, 2016; Schneider et al., 2017; Pezzoli et al., 2019; Oh et al., 2021). PDAP progression has been linked with caregiver distress, patient nursing home placement, and higher mortality leading many to consider PD a quintessential neuropsychiatric disease (Goetz and Stebbins, 1993; Carter et al., 1998). Thus, renewed efforts to understand and treat PDAP are desperately needed (Segal et al., 2021; Weintraub et al., 2022).

Prepulse inhibition (PPI) is a well-validated cross-species assay that serves as a proxy for sensorimotor gating and can be utilized to evaluate features of PDAP within PD rodent models (Swerdlow et al., 1998; Braff, 2010; Powell et al., 2012; Beaudry and Huot, 2020; Kwan et al., 2021a). Sensorimotor gating deficits are strongly indicative of cortical, basal ganglia, and brainstem dysfunction which makes it a valuable assay to detect and study neuropsychiatric changes in PD (Perriol et al., 2005; Zoetmulder et al., 2014; Lipari et al., 2023).

DA replacement with Levodopa (LD) is the current gold-standard treatment for motor symptoms of PD yet chronically induces or exacerbates motor and non-motor fluctuations (Rahman et al., 2014). One key motor fluctuation, LD-induced dyskinesia (LID), presents in up to 90% of patients and is associated with high pulsatile extracellular DA levels within the striatum (STR) and substantia nigra (SN) (Ahlskog and Muenter, 2001; Hely et al., 2005; Lindgren et al., 2010; Bhide et al., 2015). This also occurs in non-motor structures such as the hippocampus (HPC), amygdala (AMY), and prefrontal cortex (PFC), ostensibly exacerbating non-motor symptoms associated with these regions (Eskow Jaunarajs et al., 2012; Navailles et al., 2010; Diederich et al., 2015, 2016; Fabbri et al., 2017; Fridjonsdottir et al., 2021; Vinogradov et al., 2023).

Growing clinical evidence points to a positive relationship between LID and PDAP suggesting LD effects on each may share a common mechanism (Hinkle et al., 2018; Yoo et al., 2019; Luca et al., 2021; Santos-García et al., 2020). One potential neurobiological substrate is the ascending serotonin (5-HT) system, which originates in the raphe nuclei (Di Matteo et al., 2008; Gagnon and Parent, 2014). There is both clinical and preclinical support for aberrant raphe-striatal neuroplasticity contributing to LID including neuroanatomical evidence, lesion studies, and pharmacological manipulations (Eskow et al., 2009; Politis et al., 2014; Lanza and Bishop, 2018; Conti Mazza et al., 2023). These studies point to the ability of the 5-HT system to take up exogenous LD, convert it to DA, package and release it as a false neurotransmitter (Tanaka et al., 1999; Carta et al., 2007; Eskow et al., 2009; Politis et al., 2014; Sellnow et al., 2019). In non-motor regions dysregulated in PD, similar mechanisms appear to exist (Huot et al., 2012; Huot and Fox, 2013; Ohno et al., 2015; Navailles et al., 2010), though many of the experimental approaches to this point have relied on systemic pharmacological strategies, multi-target drugs or permanent lesions (Huot and Fox, 2011, 2013; Bergman et al., 2017). To directly investigate the role of the 5-HT system in LID and PDAP, we employed an acute chemogenetic approach using designer receptors exclusively activated by designer drugs (DREADDs) to selectively inhibit dorsal raphe-originating 5-HT projections to normalize forebrain input and reduce PDAP.

DREADDs are a specific strategy under the umbrella of chemogenetics whereby various G-protein-coupled receptor (GPCR) signaling pathways can be manipulated to ultimately dissect their roles in neural changes and/or behaviors in preclinical models (Campbell and Marchant, 2018; Michaelides and Hurd, 2022). The current study employed a 6-hydroxydopamine (6-OHDA) bilateral medial forebrain bundle lesion rat model to examine LD-induced complications (Castaneda et al., 1990; Moukhles et al., 1994; Paillé et al., 2007; Politis et al., 2010a, 2010b; Eskow Jaunarajs et al., 2012). This project sought to first validate previous work displaying lesion-induced motor deficits and LID development (Sakai and Gash, 1994; Ungerstedt, 1971; Truong et al., 2006; Lipari et al., 2023). Next, the selective DREADD ligand Compound 21 (C21) was employed to determine if selectively inhibiting dorsal-raphe-5-HT projections was sufficient to reduce both LID and sensorimotor gating dysfunction. In support of prior work, a positive correlation was found between LID and PDAP such that rats who expressed greater LID also showed more significant PPI deficits (Lipari et al., 2023). Notably, acute chemogenetic intervention effectively reduced established LID without impairing LD’s promotor efficacy. However, the DREADDs were ineffective for ameliorating sensorimotor gating dysfunction. Overall, these results indicate that normalizing overall raphe 5-HT output is effective for reducing LID, yet distinct 5-HT neurocircuits contributing to PDAP in a rat model of PD will require further interrogation.

## 2 Materials and methods

### 2.1 Animals

Four- to six-month-old male and female Long Evans (*N* = 34; 18M, 16F; 250–500 g) TPH2-Cre positive rats were used throughout the experiments (Envigo, Indianapolis, IN). Animals were pair housed in plastic cages (22 × 45 × 23 cm) and were given *ad libitum* access to food (Rodent Diet 5001; Lab Diet, Brentwood, MO, United States) and water. Rats were maintained on a 12/12 light/dark cycle beginning at 07:00 h in a temperature-controlled room (22–23°C). Rats were maintained in accordance with the guidelines of the Institutional Animal Care and Use Committee of Binghamton University and the “Guide for the Care and Use of Laboratory Animals” (Institute for Laboratory Animal Research, National Research Council, 2010).

#### 2.1.1 Vector production

The CRE-dependent recombinant AAV genome pAAV-hSyn-DIO-hM4D(Gi)-mCherry was obtained from Addgene (plasmid #44362; Krashes et al., 2011). It contains hM4D fused with mCherry under the control of the human synapsin promoter with the cassette flanked by lox sites. Genome was packaged into AAV9 as previously described (Sandoval et al., 2019). Briefly, genome plasmid together with the pXX6 and p9 helper plasmids were co-transfected into HEK293 cells. Three days later, viral capsids were purified from cells and media using an iodixanol gradient followed by buffer exchange. Titer was determined using ddPCR and normalized to 1 × 10^12^ viral genomes/mL using modified PBS.

### 2.2 Surgical procedures

#### 2.2.1 6-hydroxydopamine bilateral medial forebrain bundle lesion

**Figure 1:**
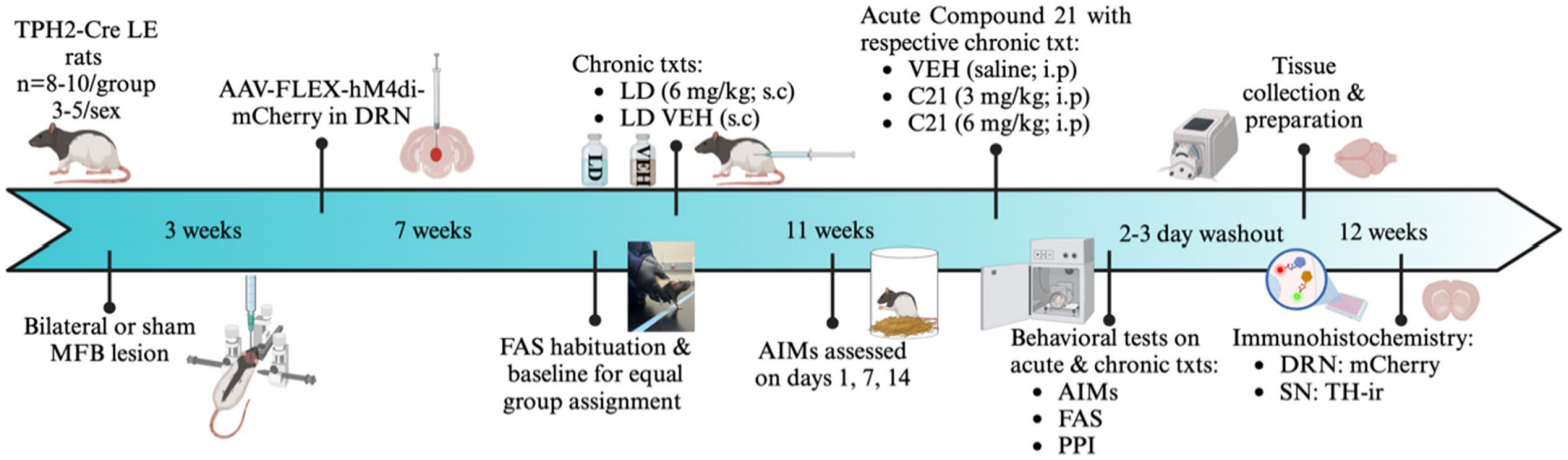
Experimental timeline and design. In all experiments, Tryptophan hydroxylase Cre recombinase positive (TPH2-Cre) Long Evans rats (*N* = 34) were given bilateral 6-hydroxydopamine hydrobromide (6-OHDA) or sham lesions of the medial forebrain bundle (MFB). After a 3-week recovery period, all rats received an infusion of AAV-DIO-hM4di-mCherry into the dorsal raphe nucleus (DRN). Animals then underwent a 4-week recovery to allow for sufficient viral transfection. All rats then went through 3 days of forepaw adjusting steps (FAS) habituation, with baseline measurement on the fourth day to determine lesion efficacy and create equally impaired groups. The following day, rats received daily L-DOPA (LD; 6 mg/kg + 15 mg/kg benserazide; s.c.) or LD Vehicle (VEH; 0.9% NaCl+0.1% ascorbic acid, s.c.) injections for a 4-week period to examine possible induction of abnormal involuntary movements (AIMs). This chronic treatment paradigm culminated in 4 between subject’s conditions [sham VEH (*n* = 8, 5M, 3F), Sham LD (*n* = 8, 4M, 4F), Lesion VEH (*n* = 8, 4M, 4F), and Lesion LD (*n* = 8, 4M, 4F)]. In the first phase of the experiment, rats were rated for AIMs on days 1, 7, and 14, where axial, limb, and orolingual (ALO) AIMs were assessed. Thereafter, in the second phase of the experiment, they received 1 of 3 acute treatments in addition to receiving their previously assigned chronic treatment: Compound 21 (C21) VEH, C21 (3 mg/kg; i.p), C21 (6 mg/kg; i.p) in a within-subjects, counterbalanced design. Altogether, there were 6 acute intervention test days with at least 24 h washout between each. After completion of experiments, allowing for at least 3-day washout, rats were transcardially perfused and stored in 30% sucrose for later collection of substantia nigra (SN), and DRN tissue for immunohistochemical analysis of mCherry and tyrosine hydroxylase (TH). Image created by BioRender.com.

Prior to surgery, all rats were handled at least 5 times to acclimate them to behavioral procedures and post-operative care. As shown in Figure 1, all rats received a bilateral DA lesion with 6-OHDA hydrobromide (6-OHDA; Sigma, St Louis, MO, United States; *n* = 18) or sham lesion with 6-OHDA vehicle (VEH; 0.9% NaCl +0.1% ascorbic acid, *n* = 16) infused bilaterally into the medial forebrain bundle (MFB), a procedure previously described in Eskow Jaunarajs et al. (2012) that produces 50–60% SNc DA cell loss and minimal DRN 5-HT loss. Briefly, rats were anesthetized with inhalant isoflurane (2–3%; Sigma) in oxygen (2.5 L/min) and placed in a stereotaxic apparatus (David Kopf Instruments, Tujunga, CA, United States) after receiving an analgesic injection of Buprenex (buprenorphine HCl; 0.03 mg/kg, i.p.; Hospira Inc., Lake Forest, IL, United States). Relative to bregma, the coordinates for injection at the MFB were as follows: AP, −1.8 mm; ML, ±2.0 mm; DV, −8.6 mm from skull, with the incisor bar positioned at 5.0 mm below the interaural line (Paxinos and Watson, 1998). A small burr hole was drilled into the skull dorsal to the injection site, and a 10 μL syringe attached to a 26-gage needle containing 6-OHDA or VEH was lowered into the MFB. Two μg of 6-OHDA dissolved in 0.9% NaCl +0.1% ascorbic acid was injected at a rate of 2 μL/min for a total volume of 2 μL on the first side. The needle was withdrawn after 5 min to allow for maximal diffusion at the injection site. This process was repeated contralaterally with a dose of 3 μg/ 2 μL. Multiple dose groups were employed to create a range of DA depletion. Sham-lesioned animals received 2 μL of 6-OHDA vehicle (0.9% NaCl +0.1% ascorbic acid) at the MFB sites. Injection concentrations were counterbalanced by side throughout the study. The surgical procedure and concentrations of neurotoxin used were determined by prior work from our laboratory which revealed that a higher dose on the second side was necessary to achieve approximately equivalent DA depletion in both hemispheres (Eskow Jaunarajs et al., 2012; Lipari et al., 2023).

#### 2.2.2 Dorsal raphe nucleus viral infusion

As shown in Figure 1, after a 3-week recovery period, all rats (*n* = 34) underwent a second survival surgery where they received an infusion of AAV-DIO-hM4Di-mCherry into the DRN (AP, −7.8 mm; ML, 0.0 mm; DV, −6.4 mm). Briefly, a 10 μL Hamilton gastight syringe was lowered at the AP and ML injection sites, while DV was lowered to −6.8 and a wait period of 1 min ensued without any infusion. Then, the needle was raised to DV −6.4 mm and the AAV-DIO-hM4Di-mCherry virus was infused at a rate of 0.5 μL/min for 4 min total culminating a total volume of 2 μL. After the infusion, the needle was held at the site for 7 min before retracting to allow for maximal diffusion after which the needle was withdrawn. All injections were administered relative to Bregma with the incisor bar positioned at 5.0 mm below the interaural line (Paxinos and Watson, 1998). All general surgical procedures were repeated as mentioned above. After both surgeries, animals were pair housed, placed in pre-warmed clean cages, and given 2 postoperative doses of carprofen (5 mg/kg, s.c.; Zoetis): one 6–12 h after surgery, and another 12 h after the first post-operative injection. Further, animals were monitored for an intensive post-operative period of 10 days where they received soft food, fruit, high calorie gel supplements, fluid replacement, and additional analgesics as needed to facilitate recovery. All experiments began 4 weeks post-viral surgery to allow for sufficient recovery time and viral transfection.

### 2.3 Experimental design

After all surgical interventions and recovery, FAS habituation and baseline was recorded to determine lesion efficacy and create equally motor-disabled groups. The following day, rats were assigned to a chronic treatment regime where they received daily injections of LD’s VEH (0.9% NaCl+0.1% ascorbic acid, s.c.) or LD methyl ester [6 mg/kg + 15 mg/kg benserazide, s.c.; Sigma], for a 4-week period (Lundblad et al., 2002; Lundblad et al., 2005). Four distinct treatment groups [Sham VEH (*n* = 8, 5M, 3F), Sham LD (*n* = 8, 4M, 4F), Lesion VEH (*n* = 8, 4M, 4F), and Lesion LD (*n* = 10, 5M, 5F)] were used for subsequent experiments. In the first phase of the study, rats were assessed for AIMs at 1, 7 and 14 days of chronic treatment. Two lesioned rats chronically treated with LD did not meet the ALO sum criteria of ≥15 and thus were excluded from further experimental manipulations.

After this 4-week chronic treatment period, all rats who met inclusion criteria entered the second phase of the investigation. Here rats received 1of 3 counterbalanced, within-subjects acute C21 treatment [C21 VEH (saline; i.p.), C21 (3 mg/kg; i.p.), C21 (6 mg/kg; i.p.)] in addition to their previously assigned LD chronic treatment. Each animal received acute and chronic treatment and underwent AIMs, FAS, and PPI testing. All drugs were administered at an injection volume of 1 mL/kg. Experimenters were blind to all experimental conditions.

### 2.4 Behavioral analyses

#### 2.4.1 Forepaw adjusting steps

The FAS test is a validated tool for measuring akinetic symptoms of PD utilized to verify lesion efficacy and monitor changes in motor performance with LD treatment (Olsson et al., 1995; Chang et al., 1999). To perform the FAS test, a trained experimenter holds each rat so that all but one forepaw are gently restrained. Rats were held at a downward angle of 80° and dragged laterally at a speed of 90 cm/10 s on each forepaw in two directions, forehand and backhand, three times each. To obtain a sum of total steps taken on each paw for each animal values were averaged into group means for total summed steps for each group (Conti et al., 2014; Conti et al., 2016). This allowed for comparison between motor deficits attributed to the lesion and motor restoration by LD and any acute treatment effects on stepping. The FAS test was also employed at post-lesion baseline to create equally motor-impaired groups to assign chronic treatments.

#### 2.4.2 Abnormal involuntary movements

Rats were monitored for LID using the abnormal involuntary movements (AIMs) rating scale that has been pharmacologically validated through the administration of known anti-dyskinetic compounds (Dekundy et al., 2007; Bishop et al., 2012; Lindenbach et al., 2015; Lindenbach et al., 2017). On AIMs days within the chronic treatment regime, directly following LD injection, rats were placed in clear plexiglass cylinders with woodchip bedding and a trained observer blind to experimental condition for rating dyskinesia 10 min after receiving LD for 1 min every 10 min consecutively for a total duration of 180 min, a procedure previously outlined in (Bishop et al., 2012; Lanza et al., 2021). For AIMs test days during our acute treatment phase, C21 was administered intraperitoneally (i.p.) 5 min before LD (6 mg/kg or VEH; s.c.) and the first rating began 10 min later. Again, AIMs were assessed for 1 min every 10 min consecutively for a total duration of 180 min. Raters incorporated modifications to this assay, detailed below, given the bilateral nature of the lesion (Paillé et al., 2004; Paillé et al., 2007). “Axial” AIMs were characterized by a dystonic twisting of the trunk (right or left) with 1 shoulder adjacent to the opposite shoulder. When expressed, it was always unilateral. “Limb” (forelimb) AIMs were defined as a purposeless and repetitive motion of either forelimb. These usually consist of subjects uncontrollably forming a fist, repetitive rhythmic jerks, or hyperextended forepaws. Lastly, “orolingual” AIMs were characterized by abnormal lateral jaw tremors and tongue protrusions. Orolingual AIMs were more subtle in the dyskinetic bilaterally lesioned rats compared those observed in the unilateral model; therefore, careful observation was required to correctly record such dyskinesia (Bishop et al., 2012). Subjects demonstrate more tongue protrusion unrelated to dyskinesia or grooming as well as dystonic movements that affect the face and could be mistaken for typical orolingual. Therefore, it was required that orolingual behaviors mentioned above were present for at least 5 consecutive seconds and unassociated with any other grooming behaviors to be classified as “true” orolingual behavior. Each behavior was ranked on a temporal scale and receive a score of: 0 (absent), 1 (present for less than 30 s), 2 (present for 30–59 s), 3 (present for the whole 60 s but interrupted by stimulus) or 4 (present for the whole 60 s and uninterruptible) (Lanza and Bishop, 2018). AIMs were observed on days 1, 7, and 14 to track potential LID development during chronic daily LD (6 mg/kg, Sigma) and observed three more times at each dose of C21 (VEH, 3 mg/kg, 6 mg/kg; HelloBio, Princeton, NJ) with animal’s previous respective chronic treatments to evaluate its effects on LID severity. Total ALO (axial, limb, and orolingual) sums were calculated for each animal. Rats that expressed an ALO sum of ≥15 were deemed dyskinetic and this was used as an exclusion criterion for lesioned animals chronically treated with LD. Two animals in this group were excluded due to failure to meet ≥15 ALO sums.

#### 2.4.3 Prepulse inhibition assay

PPI was used to measure sensorimotor gating dysfunction, a cross-species assay of psychosis (Swerdlow et al., 2001; Abbruzzese and Berardelli, 2003; Grauer et al., 2014; Kohl et al., 2013; Issy et al., 2015). The test chambers were housed in a sound attenuated cubicle with the ambient white noise level at 65 dB during all habituation and testing sessions. Habituation to the startle chambers without presentation of a startling stimulus is necessary to reduce neophobic behavior and ensure valid responses to the startle amplitude during testing (Valsamis and Schmid, 2011). Habituation occurred 3 times across 3 consecutive days with increasing session durations. On day 1, rats were placed in perforated plexiglass cylinders (20.3 × 6.4 × 8.9 cm) within the chamber for 5 min. On the 2nd and 3rd days of habituation, the duration in the chambers increased to 8 and 10 min, respectively. On each day of habituation, rats were placed into a different chamber to mimic the counterbalanced placement they would experience during testing. During test sessions, rats were placed in a perforated plexiglass cylinder (20.3 × 6.4 × 8.9 cm) on a platform that recorded the rat’s full body startle reactions, relative to the rat’s weight, in a series of classical conditioning trials (Fechter, 1974). This test measures an organism’s ability to properly regulate their auditory startle response when an acoustic predictive cue is presented. All chambers underwent a complete sound and input calibration before each testing day.

On test days, rats were placed in their respective chamber and a 5 min acclimation period ensued. Then, acclimation block 1 commenced, consisting of 10 trials presented at a fixed inter-trial interval (ITI) of 30 s. Directly following in testing block 2, animals were exposed to 7 different acoustic trials presented 10 times in a pseudorandom order for a total of 70 trials. Ten trials consisted of a 110 dB presented for 40 ms and classified as the startle stimulus. The rise and decay time consist of the time it takes for the prepulse tone to rise and fall from the maximum dB level and was 5 ms. Varying prepulse intensities of 70, 75, and 80 dB preceded the startle stimuli by 100 ms with 10 separate trials for each prepulse intensity. These 30 trials consisted of the varying prepulse intensities presented alone for a 20 ms duration. In block 2, the ITI ranged from 15 to 25 s, while the interstimulus interval was fixed at 100 ms. During all experiments, pure tones were presented at a 5 kHz frequency while background white noise (65 dB) was played continuously. Counterbalanced pharmacological treatments were administered 45 (C21 VEH, 3 mg/kg, 6 mg/kg) and 40 (LD 6 mg/kg and LD VEH) mins before subjects were placed in the chambers and acoustic testing began for a duration of 40 min. Our testing start time was determined due to evidence of peak DA levels about 40 min after LD administration (Lindgren et al., 2010). However, the half-life of LD is approximately 2 h while the PPI testing regime only lasted 40 min, therefore, animals were tested during peak DA levels (Mena et al., 2009). C21 has been shown to have a dose-dependent half-life of about 1 h with relatively rapid relief of LID; therefore, it was always administered 5 min before LD (Bonaventura et al., 2019; Jendryka et al., 2019).

### 2.5 Post-mortem analyses

#### 2.5.1 Perfusion and tissue processing

At least 3 days after final behavioral tests were run, animals were euthanized with an i.p. injection of sodium pentobarbital (200 mg/kg). They were then transcardially perfused with 120 mL of ice cold 0.9% phosphate buffered saline solution (PBS), followed by 120 mL of 4% paraformaldehyde (PFA) in PBS solution (pH = 7.4). Brains were extracted and post-fixed in PFA for 21–23 h at 4°C, and then transferred into 30% sucrose until saturated. All brains were sectioned into 40 μm coronal 1:12 series using a freezing microtome and stored in antifreeze solution at −20°C until use.

#### 2.5.2 Immunohistochemistry

A full series of free-floating sections was stained immunohistochemically for tyrosine hydroxylase (TH) (AB152, EMD Millipore, Burlington, MA) to determine lesion status; and ¼ series was stained for mCherry (600-401-379; Rockland, Massachusetts) to validate transduction (Supplementary Figure S1). The staining process is previously described in Sellnow et al. (2019). Briefly, sections were washed in 1× Tris-buffered saline (TBS) with 0.25% Triton x-100, quenched in 0.3% H_2_O_2_ for 15 min. Sections were thereafter blocked in 10% normal goat serum for 1 h and incubated in primary antibody (TH 1:4,000; mCherry 1:10,000) overnight at room temperature. On day 2, sections were incubated with the secondary antibody (biotinylated goat anti-rabbit IgG 1:500, AP132B; EMD Millipore, Burlingame, CA) for 1.5 h followed by incubation with the Vectastain Elite ABC kit (PK-6100; Vector Laboratories, Burlingame, CA) for 75 min. Sections were developed with 0.5 mg/mL 3,3′ diaminobenzidine (DAB, Sigma-Aldrich, St. Louis, MO) in 1% H_2_O_2_ working solution. Sections were mounted on glass slides, dehydrated, and coverslipped with Cytoseal (Thermo Fisher, Waltham, MA). Brightfield images were captured using a Nikon Eclipse 90i microscope. TH sections subject to artificial intelligence (AI) enumeration were acquired using a ZEISS Axioscan (ZEISS Group; Oberkochen, Germany).

#### 2.5.3 Total enumeration of TH+ neurons

**Figure 2:**
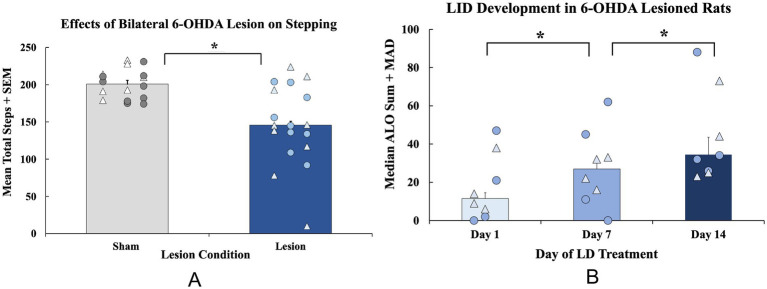
Establishment of motor deficits and development of Levodopa-induced dyskinesia (LID) in a bilateral 6-hydroxydopamine (6-OHDA) rat model of Parkinson’s disease. **(A)** The forepaw adjusting steps (FAS) test was employed 3 weeks after surgery to examine baseline motor impairments in bilateral 6-OHDA (*n* = 16; 8M, 8F) or sham (*n* = 16; 9M, 7F)—lesioned animals. FAS data are expressed as mean total steps + standard error of the mean (SEM). Baseline FAS data were analyzed with an independent-samples *t*-test (sham vs. lesion, *p < 0.05 vs. sham). **(B)** A subset of rats (*n* = 8; 4M, 4F) were administered daily LD (6 mg/kg; s.c.) for 28 days and tested for the abnormal involuntary movements (AIMs) on days 1, 7, and 14. All rats were rated for axial, limb, and orolingual (ALO) behaviors for 3 h post-injection during AIMs sessions. ALO AIMs sums are expressed as medians + median absolute deviation (MAD). Significant within-subjects AIMs differences between lesion animals were found with a non-parametric Friedman ANOVA with Wilcoxon Match pairs *post-hoc* tests (*p < 0.05 day 1 vs. day 7; day 1 vs. day 14). Individual data points represented as • Males, *Δ* Females.

Scans of TH immunoreactivity was uploaded onto the AIforia image analysis platform (AIforia Technologies; Helsinki, Finland) which uses AI deep convolutional network learning to enable quantitative histological image analysis (Penttinen et al., 2018). The SN was outlined and a custom developed deep learning process was used to enumerate the total number of TH-IR cells within the outline. Lesion severity was determined using total enumeration of TH-positive neurons in 2 representative sections within the SNc identified by presence and proximity to the fasciculus retroflexus at levels equivalent to −5.20 mm and −5.60 mm relative to bregma according to our previously validated method (Sellnow et al., 2019). Data are presented in Figure 2.

### 2.6 Statistical analyses

Baseline FAS parametric results (presented as mean total steps + standard error of the mean; S.E.M.) were assessed using an independent samples *t*-test. A 4 (Treatment condition: Baseline, C21 VEH + LD, C21(3) + LD, C21(6) + LD) × 2 (Lesion condition: sham or lesion) mixed ANOVA with Least Significant Difference (LSD) *post-hocs* was run to assess C21’s effects on stepping. AIMs data (expressed as medians + median absolute deviation; M.A.D.) were analyzed by non-parametric statistics. For within-subjects’ comparisons across days (1, 7, and 14) Friedman’s ANOVA with Wilcoxon post-hoc tests were used to analyze ALO AIMs only in LD-treated lesion animals. A second Friedman’s ANOVA with Wilcoxon post-hoc tests were run to assess the acute effects of C21 treatment (C21 VEH + LD (6 mg/kg), C21 (3 mg/kg) + LD, C21 (6 mg/kg) + LD) on dyskinesia in lesion LID animals. PPI raw data was converted into a percent PPI for every trial for each rat and was calculated with $100-\frac{\left(\mathrm{Prepulse}+\mathrm{pulse}\right)}{\mathrm{Pulse}\;\mathrm{Alone}}*100$ . In some instances, a prepulse composite score, derived from the average percent PPI score at each prepulse condition (70, 75, 80 dB) was used for analyses. When examining the joint effects of lesion and LD treatment on PPI, a 4 (chronic treatment: sham VEH, sham LD, lesion VEH, lesion LD) × 3 (Prepulse condition: 70, 75, 80 dB) mixed ANOVA was run. To analyze PPI effects as a result of acute C21 treatment in all groups, a 4 (Chronic treatment: sham VEH, sham LD, lesion VEH, lesion LD) × 3 [Acute treatment: C21 VEH, C21(3), C21(6)] repeated-measures ANOVA was used. Lastly, to assess acute effects of C21 treatment (C21 VEH + LD (6 mg/kg), C21 (3 mg/kg) + LD, C21 (6 mg/kg) + LD) on PPI in lesion LD-treated animals, a paired-samples *t*-test was employed. When rats showed an average startle response below 300, this was considered a pulse failure, and these data were excluded. TH cell counts were analyzed across all groups using a one-way ANOVA with least significant different (LSD) *post-hoc* tests. To analyze TH cell counts between collapsed sham and lesioned groups an independent samples *t*-test. Analyses for all experiments were performed using SPSS software (Chicago, IL, United States) with alpha set at *p* < 0.05.

## 3 Results

### 3.1 Establishment of motor deficits and LID in bilateral 6-OHDA lesioned rats

#### 3.1.1 FAS motor performance and LID development in bilaterally lesioned rats

**Figure 3:**
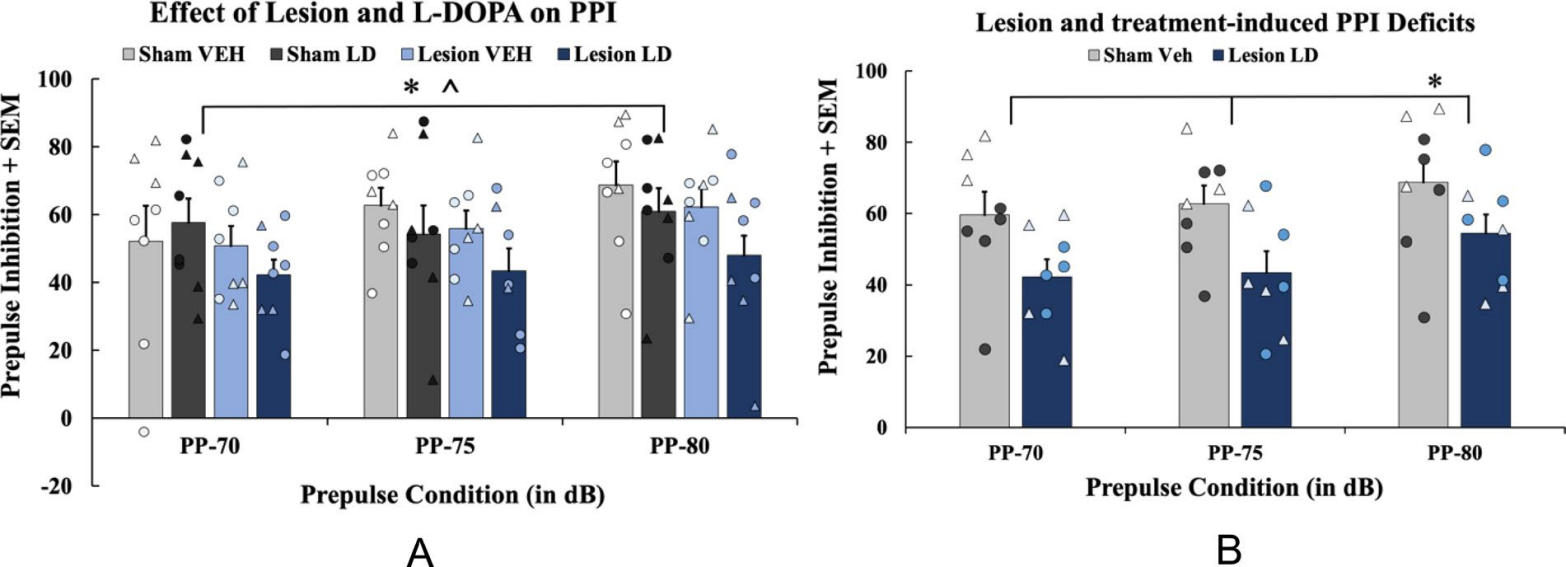
The effects of bilateral 6-hydroxydopamine (6-OHDA) lesion and history of levodopa (LD) treatment on prepulse inhibition (PPI). In a between-subjects counterbalanced design, rats were treated with daily LD Vehicle (VEH) or LD (6 mg/kg; s.c.) for 4 weeks. **(A)** To examine PPI effects as a result of chronic treatment history, a 4 (Chronic treatment: sham VEH, sham LD, lesion VEH, lesion LD) × 3 (Prepulse condition: 70, 75, 80) mixed ANOVA was used for analyses (*p < 0.05, main effect of prepulse; *p < 0.05, main effect of chronic treatment). **(B)** To test lesion and LD’s chronic effects compared to controls, PPI data from acute Compound 21 Vehicle (C21 VEH) days were analyzed with a 2 (Group: sham VEH vs. lesion LD) × 3 (Prepulse condition: 70, 75, 80 dB) mixed ANOVA with least significant difference (LSD) pairwise comparisons (*p < 0.05, prepulse 70 and 75). PPI data are presented as mean percent PPI values + standard error of the mean (SEM). Individual data points represented as • Males, Δ Females.

Sham and DA-lesioned rats were tested for baseline motor deficits using the FAS test. The following day, a 4-week chronic treatment regime with LD VEH or LD began. The AIMs test was employed on days 1, 7, and 14 to assess the development of dyskinesia. At baseline, lesioned rats displayed fewer overall steps (*t*(30) = 4.453, *p* < 0.05; *n* = 16/group, Figure 3A), indicative of DA lesion. When analyzing ALO AIMs development in lesioned, LD-treated rats, an effect of treatment day was found (*χ*^2^(2) = 10.129, *p* < 0.05; *n* = 8, Figure 3B) that Wilcoxon post-hoc analyses revealed was a result of a significant increase in AIMs from day 1 to day 7 (*p* < 0.05) that was maintained on day 14 (*p* < 0.05 vs. day 1).

#### 3.1.2 Effects of bilateral 6-OHDA lesion and history of chronic LD on PPI

**Figure 4:**
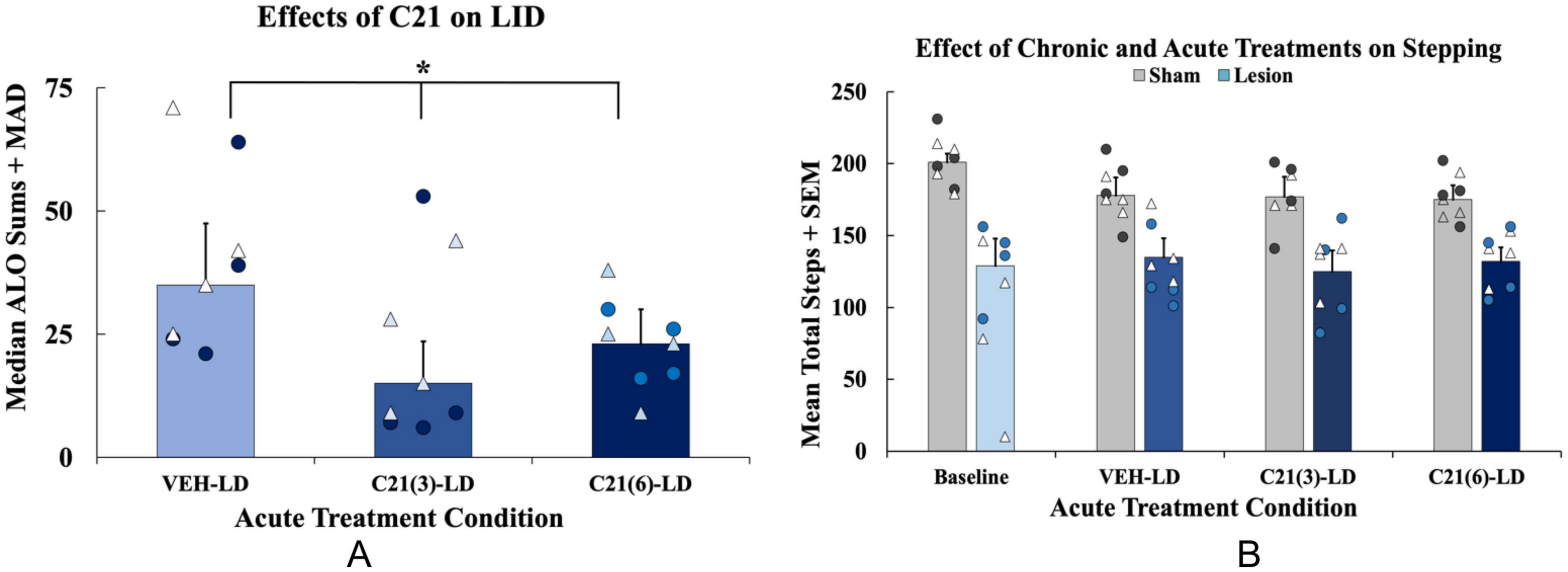
Effects of acute compound 21 (C21) on levodopa-induced dyskinesia (LID), and motor performance in levodopa (LD)-treated animals. In a within-subjects counterbalanced design rats were treated acutely with C21 vehicle (VEH) + LD, C21 (3 mg/kg; i.p.) + LD (6 mg/kg; s.c.), and C21 (6 mg/kg; i.p.) + LD (6 mg/kg; s.c.). **(A)** Abnormal involuntary movements (AIMs) were assessed for 3 h post LD-injection on all testing days. Axial, limb, and orolingual (ALO) AIMs are expressed as medians (ALO + median absolute deviation; MAD*; n* = 8; 4M, 4F). Total ALO AIMS were analyzed with non-parametric Friedman ANOVAs with Wilcoxon Match Pairs *post-hocs* (*p < 0.05 vs. VEH-LD). **(B)** Animals performed the forepaw adjusting steps (FAS) test 60 min post-LD administration on the same days that the AIMs test was performed (*n* = 8/group). FAS data are expressed as mean adjusting steps + standard error of the mean (SEM). FAS data were analyzed with a 2 [Lesion condition: lesion (*n* = 8) or sham (*n* = 8)] × 4 [Acute treatment: Baseline, C21 VEH + LD, C21(3) + LD, C21(6) + LD] mixed ANOVA with least significant difference (LSD) *post-hocs* when appropriate (*p* < 0.05 Lesion × Treatment interaction). Individual data points represented as • Males, Δ Females.

To examine effects on chronic LD treatment (*n* = 8/group), a 4 (Chronic treatment; sham VEH, sham LD, lesion VEH, lesion LD) × 3 (Prepulse condition: 70, 75, 80 dB) mixed ANOVA was employed and revealed a significant main effect of prepulse condition (*F*_(2, 58)_ = 5.994, *p* < 0.05; Figure 4A) and a main effect of chronic treatment (*F*_(3, 28)_ = 1.374, *p* < 0.05; Figure 4A). To determine if sensorimotor gating deficits emerged as a result of lesion and chronic LD treatment compared to sham controls, a 2 (Group: sham VEH or lesion LD) × 3 (Prepulse condition: 70, 75, 80 dB) mixed ANOVA was used and revealed a significant main effect of prepulse condition (*F*_(2, 28)_ = 4.167, *p* < 0.05; Figure 4B) and a main effect of group (*F*_(1, 14)_ = 5.762, *p* < 0.05). Pairwise comparisons revealed significant differences in PPI between the two groups at the 70 and 75 prepulses, respectively (*t*(14) = 2.174, *p* < 0.05; *t*(14) = 2.377, *p* < 0.05; Figure 4B) indicating PPI-related dysfunction in parkinsonian rats displaying LID.

### 3.2 Effects of acute inhibition of 5-HT raphe projections by C21 administration on LID, FAS, and PPI

#### 3.2.1 Effects of C21 on LID and motor performance

**Figure 5:**
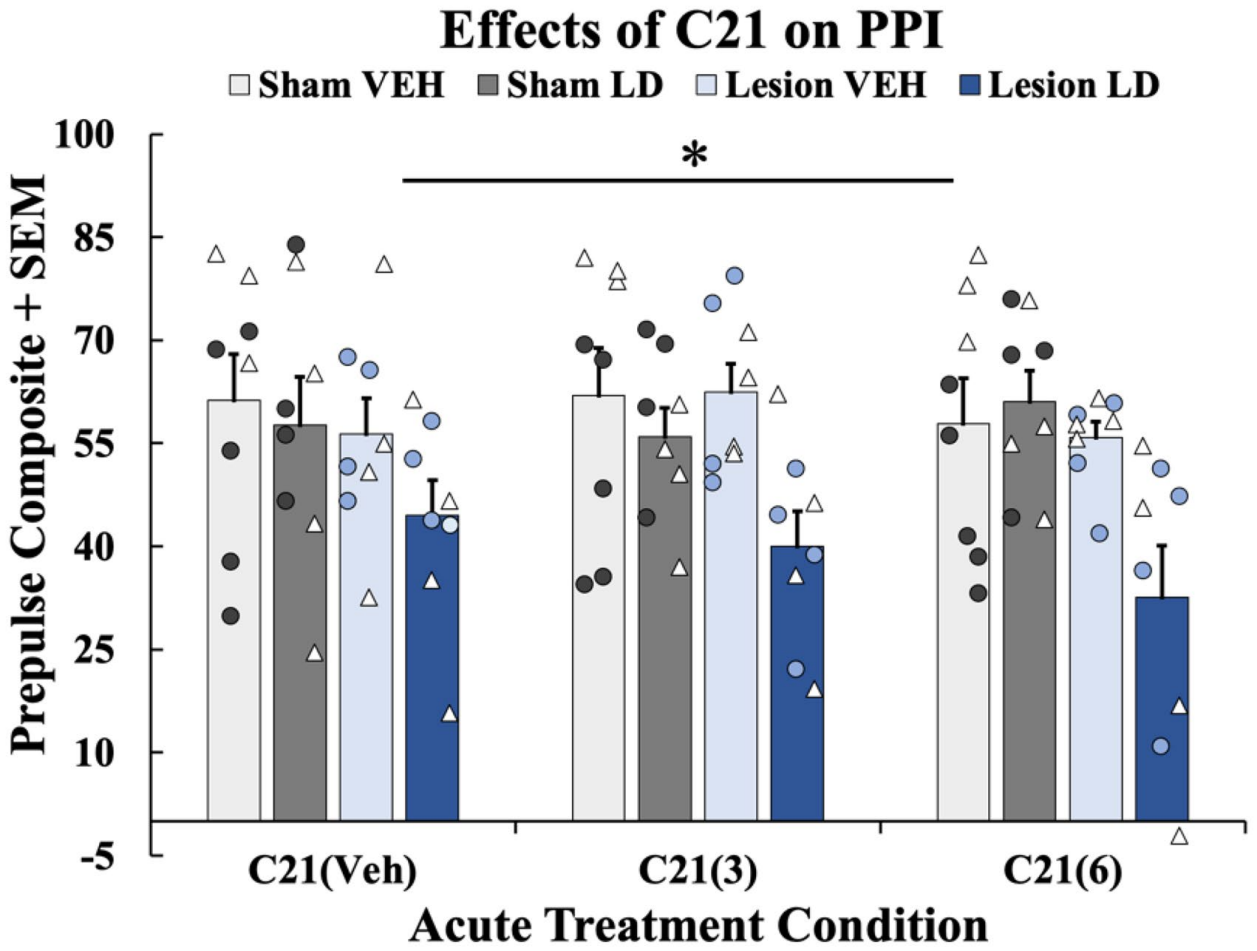
The effects of acute compound 21 (C21) treatment on prepulse inhibition (PPI). In a within-subjects counterbalanced design rats were treated acutely with Compound 21 (C21) Vehicle (VEH) + Levodopa (LD; 6 mg/kg), C21 (3 mg/kg) + LD (6 mg/kg), C21 (6 mg/kg) + LD (6 mg/kg). Rats were tested on PPI 45 min post-acute drug treatment while using their previous chronic treatment groups (sham VEH, sham LD, lesion VEH, lesion LD) as a grouping variable. A 4 (Chronic treatment: sham VEH, sham LD, lesion VEH, lesion LD) × 3 [acute treatment: C21 VEH, C21 (3 mg/kg), C21 (6 mg/kg)] mixed ANOVA was run and revealed a significant effect of chronic treatment group (*p < 0.05). Least significant difference (LSD) *post-hocs* suggested lesion LD animals showed significantly impaired PPI at all doses on C21 compared to all other chronic groups (*p < 0.05). PPI data are presented as mean percent PPI values + standard error of the mean (SEM). Individual data points represented as • Males, Δ Females.

Rats were tested across 3 treatment days to examine the effects of C21 on AIMS and FAS stepping. Rats received C21 VEH, C21 (3 mg/kg), and C21 (6 mg/kg) in addition to their respective chronic treatment [LD VΕΗ or LD (6 mg/kg)] on each testing day. ALO AIMs were analyzed in lesion LD-treated rats only using a non-parametric Friedman ANOVA revealing a significant effect of acute treatment (*χ*^2^(2) = 11.400, *p* < 0.05; *n* = 8, Figure 5A). Wilcoxon post-hocs demonstrated that rats displayed significantly lower ALO AIMs expression when treated with C21 (3 or 6 mg/kg) + LD, compared to C21 VEH group (*p* < 0.05; Figure 5A). For motor performance on the FAS test, a 2 (Lesion condition: lesion vs. sham) × 4 (Acute treatment: Baseline, C21 VEH + LD, C21 (3 mg/kg) + LD, C21 (6 mg/kg) + LD) mixed ANOVA revealed a significant Lesion x Treatment interaction (*F*_(3, 42)_ = 3.791, *p* < 0.05, Figure 5B).

#### 3.2.2 Effects of C21 on PPI

**Figure 6:**
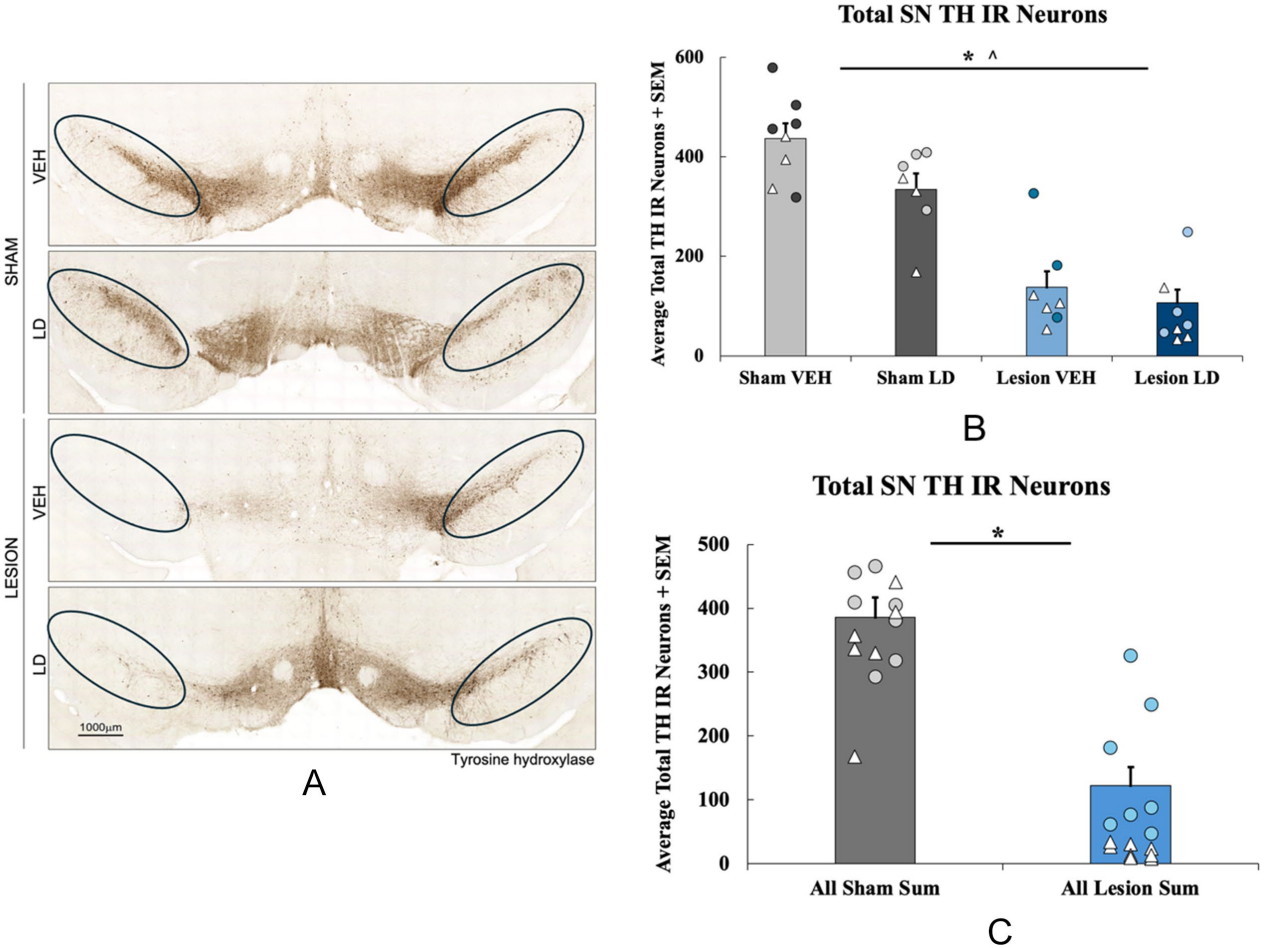
Loss of tyrosine hydroxylase (TH) immunoreactive neurons following bilateral 6-hydroxydopamine (6-ODHA) lesion and chronic treatment. **(A)** Representative images of TH immunoreactivity of nigral sections from each experimental group with circles indicating where cells were counted for each slice. **(B)** Total TH neuron loss quantified in the substantia nigra (SN) between sham and lesion rats either treated chronically with levodopa (LD) or vehicle (VEH; *p < 0.05 lesion condition; *p < 0.05 chronic treatment). **(C)** Total TH neuron loss quantified in the SN between all sham and lesion groups collapsed across chronic treatments. (*p < 0.05 vs. Sham VEH). Individual data points represented as • Males, Δ Females.

To discern whether acute 5-HT raphe inhibition could modify sensorimotor gating, 3 PPI test days occurred in the presence of C21 or its vehicle. A 4 (Chronic treatment: sham VEH, sham LD, lesion VEH, lesion LD) × 3 [Acute treatment: C21 VEH, C21 (3 mg/kg), C21 (6 mg/kg)] repeated-measures ANOVA was employed. Analyses revealed a significant main effect of chronic treatment group (*F*_(3, 28)_ = 4.443, *p* < 0.05; Figure 6). Further LSD post-hoc tests revealed PPI was significantly impaired in the lesion LD group compared to all other conditions further supporting lesion and chronic LD-induced PPI deficits not rescued by C21 (*p* < 0.05; Figure 6).

### 3.3 Post-mortem analyses

#### 3.3.1 Effects of bilateral 6-OHDA lesion on TH positive cells in the SNc

The effects of the bilateral 6-OHDA lesion were examined using immunohistochemistry followed by total enumeration of nigral tyrosine hydroxylase immunoreactive neurons (TH; Figure 2A). A 2 (lesion condition: sham or 6-OHDA) × 2 (chronic treatment: LD VEH or LD) ANOVA revealed a significant main effect of lesion condition (*F*_(1, 28)_ = 83.251, *p* < 0.05; Figure 2B) and a main effect of chronic treatment (*F*_(1, 28)_ = 6.040, *p* < 0.05; Figure 2B) for TH levels. When treatment groups were collapsed across sham and lesion conditions, an independent samples *t*-test showed a significant 50% reduction in TH+ cells in lesion subjects compared to the sham group (*t*_(30)_ = 8.774, *p* < 0.05; Figure 2C).

#### 3.3.2 Validation of transduction

All subjects were assessed for mCherry immunoreactivity in the DRN to validate viral transduction (Supplementary Figure S1).

## 4 Discussion

The current investigation studied the motor and non-motor effects of bilateral 6-OHDA or sham lesions, motor and non-motor side effects of chronic LD, and, for the first time, chemogenetics, to test the involvement of the 5-HT system in their expression. With regard to motor features, rats with bilateral MFB 6-OHDA lesions developed significant motor impairments and modeled signs of akinesia (Figure 3A), corroborating previous findings (Olsson et al., 1995; Chang et al., 1999; Lipari et al., 2023). Moreover, animals that were chronically treated with LD developed moderate and stable LID (Figure 3B) representative of the clinical side effects experienced by a subset of patients after long-term LD treatment (Ahlskog and Muenter, 2001; Connolly and Lang, 2014). Importantly, the inhibitory DREADDs intervention working ostensibly in the raphe-striatal circuit, significantly reduced LID in lesion LD animals (Figure 5A) while having no effects on stepping abilities in any group (Figure 5B). While lesion LD-treated animals showed significant PPI deficits compared to all other groups, C21 was unable to reverse this deficiency in sensorimotor gating (Figure 6). Our post-mortem data support our behavioral effects showing significant TH loss in lesion animals (Figure 2) and sufficient transduction within the DRN to support the efficacy of our DREADDs intervention (Supplementary Figure S1).

Accumulated evidence has shown that at least 60% striatal DA loss in rodent models is required for producing LID (Schneider, 1989; Pearce et al., 1995; Di Monte et al., 2000; Winkler et al., 2002; Cenci, 2014; Engber et al., 1991; Ishida et al., 2000; Flores et al., 2023). While we did not examine terminal changes in DA, previous high performance liquid chromatography data from our lab corroborate this idea showing around 60% terminal DA loss in a 6-OHDA bilateral MFB lesion model (Lipari et al., 2023). That said, the current findings did uncover some variability in LID that was associated with TH cell loss, similar to prior work with this model (Lipari et al., 2023; Figure 2). While not quantified, it is important to note that LID in a bilateral model presents as both choreic and dystonic movements which requires further attention in future studies employing this model. This varying severity and expression of LID in our animals may recapitulate the heterogeneity of PD patients, where only some individuals display LID and only a subset experience severe LID (Cenci et al., 2020; Kim et al., 2020). In this regard, the bilateral 6-OHDA MFB lesion model may help elucidate the more precise mechanisms for why not every patient experiences LID with chronic LD treatment and potential neuroprotective and/or interventional strategies for avoiding the negative side effects of long-term therapies.

The use of a bilateral 6-OHDA MFB lesion model also provided us with a unique opportunity to mimic PD non-motor deficits and in particular, LD related side effects such as PDAP (Pellicano et al., 2007; Postuma et al., 2012). Prior literature has been mixed as to whether PDAP is driven by disease-associated DA loss or is treatment-dependent (Weiner et al., 2000; Zahodne and Fernandez, 2008; Fénelon and Alves, 2010; Schneider et al., 2017). While the association between DA loss and sensorimotor dysfunction in PD has not been adequately characterized (Swerdlow et al., 2000; Perriol et al., 2005), clinical data has revealed that within later PD stages, PDAP is positively related to LID expression and severity (Cenci, 2014; Yoo et al., 2019; Luca et al., 2021). Recent findings from our lab corroborate this showing that lesioned rats with the greatest LID also showed significant sensorimotor gating deficits (Lipari et al., 2023) matching the clinical literature (Yoo et al., 2019; Santos-García et al., 2020; Luca et al., 2021). The current work further supported this, demonstrating that lesioned animals chronically treated with LD show the greatest deficits on PPI (Figures 4A,B). Taken together, more severe DA depletion in tandem with chronic LD treatment may be necessary to elicit sensorimotor gating dysfunction (Vuillermot et al., 2011; Bleickardt et al., 2012; Issy et al., 2015; Factor et al., 2017; Lipari et al., 2023).

LID development and maintenance has reliably been attributed to maladaptive neuroplasticity within the serotonergic raphe-striatal circuit (Brown and Molliver, 2000; Carta et al., 2007; Eskow et al., 2009; Manfredsson et al., 2014; Politis et al., 2014; Sellnow et al., 2019). Indeed, DRN 5-HT neurons possess the machinery to convert exogenous LD to DA and in states of DA depletion or denervation subsequently release it in affected regions (Kannari et al., 2006; Politis et al., 2014; Rylander et al., 2010; Brown and Molliver, 2000; Tanaka et al., 1999; Lindgren et al., 2010; Fu et al., 2018). While this compensation may initially be helpful, the terminal DA transporter and presynaptic D2 receptor is virtually absent in later stage PD patients and since 5-HT cells lack autoregulatory mechanisms for DA, its unregulated release may manifest as unwanted side effects such as LID and PDAP (Calabresi et al., 2015; Yoo et al., 2018; Sellnow et al., 2019). Given the extensive clinical and preclinical support for this mechanism of LID, we hypothesized that DREADD infusion into the DRN in TPH2-Cre + rats would selectively inhibit 5-HT-raphe-projections and normalize both LID and sensorimotor gating dysfunction.

As predicted, the selective DREADD ligand C21 at both doses significantly reduced LID in lesioned animals chronically treated with LD (Figure 5A) while having no effects on stepping in any group (Figure 5B). Of note, while the FAS test, often used for unilaterally lesioned rats, was sensitive enough to detect lesion-induced deficits in stepping in bilateral lesioned rats, here it was not sensitive enough to detect treatment-related improvements with LD (Olsson et al., 1995; Chang et al., 1999). Interestingly, the lower dose of C21 showed greater efficacy in attenuating ALO AIMs expression (Figure 5A). This finding is suggestive of the dose-dependency of C21, whereby higher doses may lose some degree of selectivity (Goutaudier et al., 2020). Our C21 effects are in line with previous pharmacological and neurochemical data which suggests dampening striatal DA release from 5-HT neurons through 5-HT_1A_ autoreceptor activation in DRN significantly reduces and LID development and expression (Dupre et al., 2007; Meadows et al., 2018; Smith et al., 2022; Altwal et al., 2020, 2021; Depoortere et al., 2020; Budrow, 2023; Budrow et al., 2023). Indeed, when you block 5-HT_1A_ with selective antagonists, 5-HT_1A_ agonists’ anti-dyskinetic effects are significantly attenuated (Bishop et al., 2009; Eskow et al., 2009; Altwal et al., 2021; Budrow et al., 2023).

When examining PDAP, using PPI, we found no lesion-induced deficits, but chronic LD-treated, lesioned rats displayed sensorimotor gating deficits compared to all other groups (Figure 4). Interestingly, despite improvements seen with acute C21 treatment on LID expression, this strategy did not lead to reversal in PPI deficits within lesion LD-treated animals (Figure 6). This suggests that omnibus suppression of DRN and its projections with C21 was limited in its ability to reduce sensorimotor dysfunction. To date, research supports the sensorimotor gating circuit in rodents as including the limbic cortex, striatum, pallidum, and pontine tegmentum (Kodsi and Swerdlow, 1995; Li et al., 2009). PPI more so reflects a more central behavioral gating process mediated by forebrain circuitry (Swerdlow et al., 2000). Given the dissociation of C21 effects on LID vs. PPI, in the LD-treated, parkinsonian brain, 5-HT fibers originating in the DRN may more heavily innervate motor-associated regions rather than structures primarily involved in sensorimotor gating (Azmitia and Segal, 1978; Hensler et al., 1994), though this remains an open question since 5-HT neurons project to a plethora of non-motor regions including the nucleus accumbens (NAc), medial prefrontal cortex (mPFC), and amygdala nuclei that receive significant DA innervation (Azmitia and Segal, 1978; Steinbusch, 1984; Van Bockstaele et al., 1993). In fact, given that novel therapies alleviating PDAP mediate DA, 5-HT and glutamatergic transmission ostensibly within the mPFC, specific suppression or even activation of forebrain circuits may be necessary to alter PPI deficits (Huot and Fox, 2011, 2013; Nuara et al., 2020, 2021, 2022; Kwan et al., 2021b). Importantly, the lateral habenula receives inputs from both the basal ganglia and the limbic circuit while sending efferents to regions densely innervated by DA and 5-HT (Hikosaka, 2010; Cools et al., 2008; Lecourtier et al., 2007). Thus, this could be an important region we did not investigate involved in both motor and non-motor functions that may have been affected by mediating DRN transmission (Proulx et al., 2014). This is further hinted by findings that nicotine infusions into the lateral habenula dose-dependently attenuates amphetamine-induced PPI deficits implicating this structure in features of sensorimotor gating (Larrauri et al., 2015). Thus, our DREADD approach may either have been too specific, by influencing only DRN 5-HT or may have not been specific enough to target the essential circuits for the expression of PDAP.

## 5 Limitations

Although this study provides new insight into motor and non-motor-related LD side effects, there remain several unanswered questions. For example, while our chemogenetic approach is a unique and selective genetic tool to investigate circuit-specific questions, the strategy used here may not have fully engaged the primary region(s) involved in PDAP. In our analyses of DREADD transduction, while we found mCherry expression in several regions including the DRN and those innervated by DRN, supporting our technical approach (Supplementary Figure S1), yet it is not entirely clear what level of expression is required to alter terminal neurotransmission. On one hand, our expression in dorsal striatum strongly supports C21 suppression of LID, the first report of such effects using chemogenetics and in line with lesion, pharmacology, and genetic studies (Carta et al., 2007; Eskow et al., 2009; Bishop et al., 2009; Huot et al., 2015; Lanza and Bishop, 2018; Sellnow et al., 2019). However, mCherry expression in regions involved in PDAP like ventral striatum, pallidum, and mPFC while detected, was not defined as an *a priori* inclusion criterion, nor analyzed for each subject. Therefore, we expect that our findings may not fully represent the extent to which DRN influences the PPI response. Moreover, as mentioned above, while we were able to confine DREADD expression to 5-HT cells of the DRN, any 5-HT projections from DRN cells could have been suppressed, possibly with opposing effects. Future work using transactional viral strategies will allow us to isolate neurocircuits that may uniquely contribute to motor vs. non-motor phenotypes (Fenno et al., 2014). Finally, it is worth mention that synucleinopathies may also influence the occurrence of non-motor symptoms and fluctuations, thus future projects should consider synuclein models to capture aspects of PD not possible with a neurotoxin approach (Jellinger, 2011, 2017; Gubinelli et al., 2022a, 2022b).

## 6 Conclusion

This work employed unique and specific genetic tools to selectively target aberrant neuroplasticity within 5-HT raphe-projecting circuits and ameliorate late-stage treatment-induced complications such as LID and PDAP. Here we found evidence of a dissociation between the contributions of 5-HT raphe-projecting cells in LID, which was suppressed by circuit inhibition, and PDAP which was unaffected. These intriguing findings implicate a formidable though complex role for the 5-HT system in PD, the continued study of which could help to optimize treatments and significantly improve the quality of life for millions of patients worldwide.

## Data Availability

The raw data supporting the conclusions of this article will be made available by the authors, without undue reservation.

## Supplementary material

Supplementary Figure S1 DREADDs transduction in the dorsal raphe nucleus (DRN). All subjects were assessed for mCherry immunoreactivity in the DRN. **(A)** Representative mCherry immunoreactivity in the DRN. **(B)** Transgene expression was visualized with mCherry staining in the DRN.
