# Corrigendum: The effects of chemogenetic targeting of serotonin-projecting pathways on L-DOPA-induced dyskinesia and psychosis in a bilateral rat model of Parkinson's disease

**DOI:** 10.3389/fncir.2025.1593235

**Published:** 2025-04-01

**Authors:** Natalie Lipari, Ashley Galfano, Shruti Venkatesh, Han Grezenko, Ivette M. Sandoval, Fredric P. Manfredsson, Christopher Bishop

**Affiliations:** ^1^Department of Psychology, Binghamton University, Binghamton, NY, United States; ^2^Barrow Neurological Institute, Phoenix, AZ, United States

**Keywords:** PDAP, DREADDs, 5-HT, LID, bilateral, PD

In the published article, there was an error in the legend for Figure 2 as published. The corrected legend for Figure 2 appears below.

“**Figure 2**. Establishment of motor deficits and development of Levodopa-induced dyskinesia (LID) in a bilateral 6-hydroxydopamine (6-OHDA) rat model of Parkinson's disease. **(A)** The forepaw adjusting steps (FAS) test was employed 3 weeks after surgery to examine baseline motor impairments in bilateral 6-OHDA (*n* = 16; 8 M, 8 F) or sham (*n* = 16; 9 M, 7 F) -lesioned animals. FAS data are expressed as mean total steps + standard error of the mean (SEM). Baseline FAS data were analyzed with an independent-samples *t*-test (sham vs. lesion, ^*^p < 0.05 vs. sham). **(B)** A subset of rats (*n* = 8; 4 M, 4 F) were administered daily LD (6 mg/kg; s.c) for 28 days and tested for the abnormal involuntary movements (AIMs) on days 1, 7, and 14. All rats were rated for axial, limb, and orolingual (ALO) behaviors for 3 h post-injection during AIMs sessions. ALO AIMs sums are expressed as medians + median absolute deviation (MAD). Significant within-subjects AIMs differences between lesion animals were found with a non-parametric Friedman ANOVA with Wilcoxon Match pairs *post-hoc* tests (^*^p < 0.05 day 1 vs. day 7; day 1 vs. day 14). Individual data points represented as • males, Δ females.”

In the published article, there was an error in the legend for Figure 3 as published. The corrected legend for Figure 3 appears below.

**“Figure 3**. The effects of bilateral 6-hydroxydopamine (6-OHDA) lesion and history of levodopa (LD) treatment on prepulse inhibition (PPI). In a between-subjects counterbalanced design, rats were treated with daily LD Vehicle (VEH) or LD (6 mg/kg; s.c.) for 4 weeks. **(A)** To examine PPI effects as a result of chronic treatment history, a 4 (Chronic treatment: sham VEH, sham LD, lesion VEH, lesion LD) × 3 (Prepulse condition: 70, 75, 80) mixed ANOVA was used for analyses (^*^p < 0.05, main effect of prepulse; ^*^p < 0.05, main effect of chronic treatment). **(B)** To test lesion and LD's chronic effects compared to controls, PPI data from acute Compound 21 Vehicle (C21 VEH) days were analyzed with a 2 (Group: sham VEH vs. lesion LD) × 3 (Prepulse condition: 70, 75, 80 dB) mixed ANOVA with least significant difference (LSD) pairwise comparisons (^*^p < 0.05, prepulse 70 and 75). PPI data are presented as mean percent PPI values + standard error of the mean (SEM). Individual data points represented as • males, Δ females.”

In the published article, there was an error in the legend for Figure 4 as published. The corrected legend for Figure 4 appears below.

**“Figure 4**. Effects of acute compound 21 (C21) on levodopa-induced dyskinesia (LID), and motor performance in levodopa (LD)-treated animals. In a within-subjects counterbalanced design rats were treated acutely with C21 Vehicle (VEH) + LD, C21 (3 mg/kg; i.p.) + LD (6 mg/kg; s.c.), and C21 (6 mg/kg; i.p.) + LD (6 mg/kg; s.c.). **(A)** Abnormal involuntary movements (AIMs) were assessed for 3 h post LD-injection on all testing days. Axial, limb, and orolingual (ALO) AIMs are expressed as medians (ALO + median absolute deviation; MAD; *n* = 8; 4 M, 4 F). Total ALO AIMS were analyzed with non-parametric Friedman ANOVAs with Wilcoxon Match Pairs *post-hocs* (^*^p < 0.05 vs. VEH-LD). **(B)** Animals performed the forepaw adjusting steps (FAS) test 60 min post-LD administration on the same days that the AIMs test was performed (*n* = 8/group). FAS data are expressed as mean adjusting steps + standard error of the mean (SEM). FAS data were analyzed with a 2 [Lesion condition: lesion (*n* = 8) or sham (*n* = 8)] × 4 [Acute treatment: baseline, C21 VEH + LD, C21(3) + LD, C21(6) + LD] mixed ANOVA with least significant difference (LSD) *post-hocs* when appropriate (*p* < 0.05 lesion × treatment interaction). Individual data points represented as • males, Δ females.”

In the published article, there was an error in the legend for Figure 5 as published. The corrected legend for Figure 5 appears below.

“**Figure 5**. The effects of acute compound 21 (C21) treatment on prepulse inhibition (PPI). In a within-subjects counterbalanced design rats were treated acutely with Compound 21 (C21) Vehicle (VEH) + Levodopa (LD; 6mg/kg), C21 (3mg/kg) + LD (6 mg/kg), C21 (6 mg/kg) + LD (6 mg/kg). Rats were tested on PPI 45 min post-acute drug treatment while using their previous chronic treatment groups (sham VEH, sham LD, lesion VEH, lesion LD) as a grouping variable. A 4 (Chronic treatment: sham VEH, sham LD, lesion VEH, lesion LD) × 3 [acute treatment: C21 VEH, C21(3mg/kg), C21(6mg/kg)] mixed ANOVA was run and revealed a significant effect of chronic treatment group (^*^p < 0.05). Least significant difference (LSD) *post-hocs* suggested lesion LD animals showed significantly impaired PPI at all doses on C21 compared to all other chronic groups (^*^p < 0.05). PPI data are presented as mean percent PPI values + standard error of the mean (SEM). Individual data points represented as • males, Δ females.”

In the published article, there was an error in the legend for Figure 6 as published. The corrected legend for Figure 6 appears below.

“**Figure 6**. Loss of tyrosine hydroxylase (TH) immunoreactive neurons following bilateral 6-hydroxydopamine (6-ODHA) lesion and chronic treatment. **(A)** Representative images of TH immunoreactivity of nigral sections from each experimental group with circles indicating where cells were counted for each slice. **(B)** Total TH neuron loss quantified in the substantia nigra (SN) between sham and lesion rats either treated chronically with levodopa (LD) or vehicle (VEH; ^*^p < 0.05 lesion condition; ^*^p < 0.05 chronic treatment). **(C)** Total TH neuron loss quantified in the SN between all sham and lesion groups collapsed across chronic treatments (^*^p < 0.05 vs. Sham VEH). Individual data points represented as • males, Δ females.”

The authors apologize for these errors and state that they do not change the scientific conclusions of the article in any way. The original article has been updated.

