# Supplementary figures and images for: The effects of chemogenetic targeting of serotonin-projecting pathways on L-DOPA-induced dyskinesia and psychosis in a bilateral rat model of Parkinson’s disease
Source: Front Neural Circuits. 2024 Nov 14;18:1463941. doi: 10.3389/fncir.2024.1463941 (PMC11615880; doi:10.3389/fncir.2024.1463941)

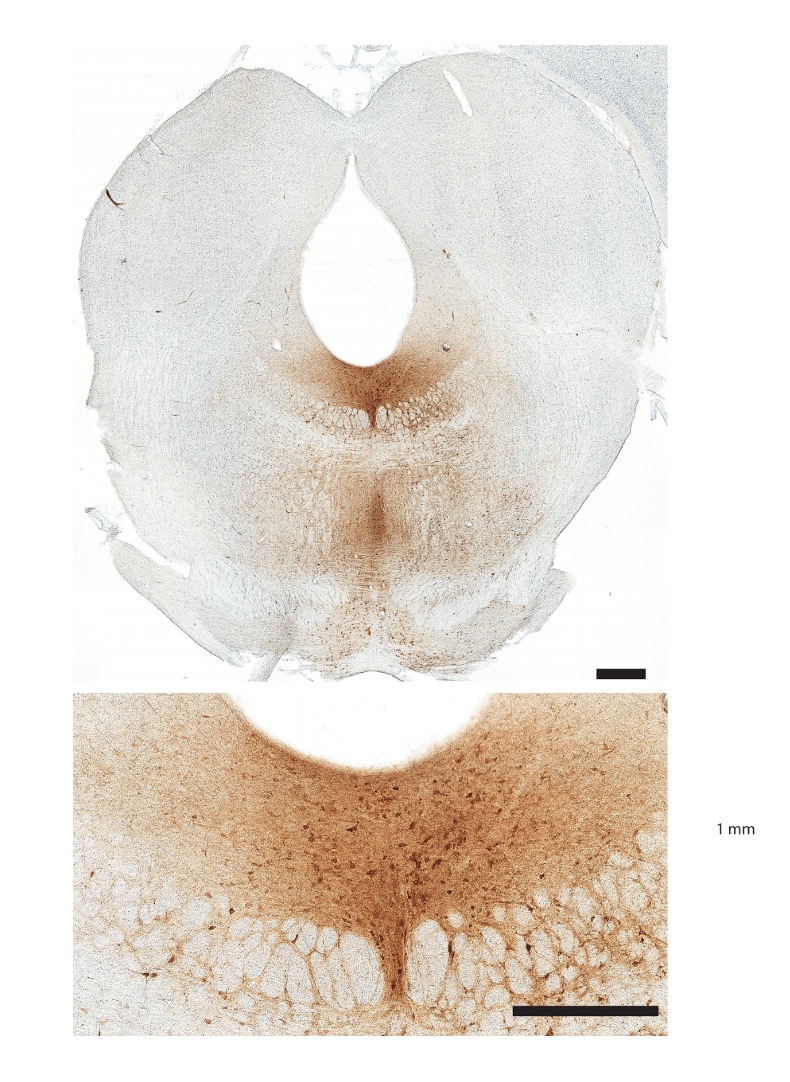

Supplement: Supplementary Figure S1 — DREADDs transduction in the dorsal raphe nucleus (DRN). All subjects were assessed for mCherry immunoreactivity in the DRN. (A) Representative mCherry immunoreactivity in the DRN. (B) Transgene expression was visualized with mCherry staining in the DRN. [file Image_1.JPEG]
